# Natural product based composite for extraction of arsenic (III) from waste water

**DOI:** 10.1186/s13065-017-0261-9

**Published:** 2017-04-12

**Authors:** N. Akartasse, E. Mejdoubi, B. Razzouki, K. Azzaoui, S. Jodeh, O. Hamed, M. Ramdani, A. Lamhamdi, M. Berrabah, I. Lahmass, W. Jodeh, S. El Hajjaji

**Affiliations:** 1 Laboratory LMSAC, Faculty of Sciences, Mohamed 1st University, P.O. Box 717, 60000 Oujda, Morocco; 2 Department of Chemistry, LS3ME, Faculty of Sciences, University Mohammed V, Rabat, Morocco; 3 Department of Chemistry, An-Najah National University, P.O. Box 7, Nablus, Palestine; 4 Laboratory LCAE-URAC18, Faculty of Sciences, Mohamed 1st University, 60000 Oujda, Morocco; 5 National School of Applied Sciences Al Hoceima, Mohamed 1st University, P.O. Box 717, 60000 Oujda, Morocco; 6 Laboratory of Biochemistry Faculty of Sciences, Mohamed 1st University, P.O. Box 717, 60000 Oujda, Morocco; 7 Deapartment of Human Medicine, An-Najah National University, P. O. Box 7, Nablus, Palestine

**Keywords:** Hydroxyapatite, Gum Arabic, Composite, Arsenic, Adsorption, Kinetic

## Abstract

Natural based composites of hydroxyapatite/Gum Arabic designed for removal of toxic metal arsenic (III) from waste water were synthesized and evaluated. Several composites with various compositions were prepared by the wet chemical method and analyzed using various spectroscopic and analytical methods such as: Fourier transform infrared spectroscopy, total organic carbon production, XRD analysis and scanning electron microscope. The rates of weight loss and water absorption of the HAp/GA composites as a function of time were evaluated in phosphate-buffered saline solution at 37 °C and a pH of 7.4. The effects of several variables on adsorption of arsenic (III) by HAp/GA composites were evaluated. The variables include arsenic (III) concentration, contact time (t) and complex surface nature of HAp/GA composite. Three surface complexation models were used to study the mechanisms controlled the adsorption. The models were Langmuir, Freundlich and Dubinin Radushkevich. The adsorption kinetic of arsenic (III) on the composite surface was described by three modes: pseudo first order, pseudo second order and the intra particle diffusion. The results revealed that, the rate of adsorption of arsenic (III) by HAp/GA composites was controlled by two main factors: the initial concentration of arsenic (III) and the contact time. The kinetic studies also showed that, the rate of adsorption is a second order. The results indicate that, composite offered in this study could be a valuable tool for removing toxic metals for contaminated water by adsorption.

**Graphical abstract:**
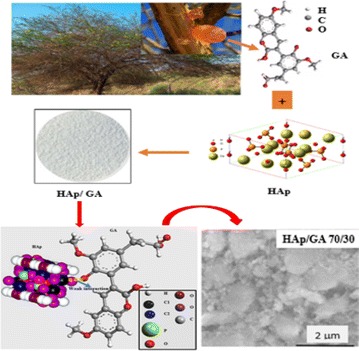
.

.

## Background

In recent years, there has been an increasing concern of environmental pollution and public health issues associated with heavy metals. Sources of heavy metals has risen dramatically to include mining, industrial, medical, agricultural, household chemicals, and others [1]. Among the metal that raise serious concerns are Hg, Cr, Ni, Zn, Cu, AS, and Cd [2].

The main source of the heavy metals in wastewaters are industrial discharges and household chemical.

Heavy metals in the ground and waste water are usually present in the form of inorganic complexes. The complexes ligands are unlikely to be organic, as they are non-biodegradable.

Several processes for removing heavy metals from waste water have been developed. Among these are chemical electrode solvent extraction, ion-exchange, activated carbon adsorption, precipitation and adsorption [3, 4]. The adsorption received the highest attention since it is simple, inexpensive, and effective especially in wastewater [5, 6].

Nanotechnology is one of the most promising techniques for metal removal from waste water. Nanoparticles have high surface area to volume ratio which provides optimum kinetics for metal binding [7, 8].

Among the above mentioned toxic heavy metals, arsenic has received the most attention and concern, because it is highly toxic and cause chronic effects on human health [9–11]. Arsenic presents in four oxidation states −3, 0, +3 and +5. The most abundant forms of arsenic in soil and waste water are with +3 and +5 oxidation states. An example of As(V) is H_3_AsO_4_ and of AS(III) is H_3_AsO_3_ [12]. Inorganic arsenic compounds are more toxic than organic arsenic ones, and As(III) is more toxic than As(V) [13]. Environment contamination of arsenic mainly comes from production and use of pesticides and other materials such as glass, paper and semiconductors. Pesticides are considered the major source of arsenic compounds in wastewater and ground. Examples on these pesticides are disodium methane arsenate (DSMA), lead arsenate, Ca_3_AsO_4_, monosodium methane arsenate (MSMA), copper acetoarsenite, cacodylic acid (used in process of cotton production) and arsenic acid (H_3_AsO_4_) [14, 15].

The major concern aroused when high concentrations of arsenic was detected in the ground and surface water at several regions of the world, including India, Bangladesh, Taiwan, Chile, Western United States, and Vietnam [16]. Several methods are known to be effective in removing arsenic such as: coagulation, precipitation, chromatography, adsorption, and co-precipitation. The adsorption is process involves the adsorption of arsenic on alumina and active carbon [16]. Adsorption process is the most effective and most widely used. Since, low cost materials such as hydroxyapatite, clay, agricultural residues and activated charcoal are used in this process [17].

Recently, several publications showed the possibility of using calcium phosphates hydroxyapatite (HAp) biomaterials composites as an adsorbent for heavy metals [18–21] and residual pesticides [22] from water and land. It was chosen because of is has highly porous structure. Unfortunately, it was found that, HAp has low adsorption capacity for metal, this was attributed to the limited number of coordination sites on HAp. So the use of HAp as a metal adsorbent was very limited. Its highly porous structure makes it unique and attractive for. One approach taking advantage of its highly porous structure and enhancing its adsorbent efficiency for metals is by blending it with a material that has good chemical affinity for hydroxyapatite and metals. Gum Arabic was chosen for this purpose.

Gum Arabic (GA) is a mixture of polysaccharides and inorganic salts. The inorganic salts composed of calcium, magnesium and potassium. The polysaccharide part composed of a skeleton and side chains. The skeleton consist of the repeat unit β-d-galactopyranosyl 1.3 and the side chains are composed of two five units of β-d-galactopyranosyl 1.3, that are attached to the main chain by 1.6 links. Gum Arabic (GA) is a well-known natural material with large number of applications. It is widely used in the pharmaceutical, cosmetic and food industries. It was also used as an emulsifier and stabilizer. In some developing countries GA is used to treat chronic kidney disease [23].

Recently, the use of GA has been extended to the nanotechnology and nanomedicine fields. Since it is biocompatible for in vivo applications and can stabilize the nanostructures. The branching and its high contents of galactose makes it interacts well with the asialoglyco protein receptors of hepatocytes. GA has been probed for coating and increasing the biocompatibility (in vitro and in vivo studies) of iron oxide magnetic nanoparticles [24], gold nanoparticles [25], carbon nanotubes [26] and quantum dot nanocolloids [27].

In this work various composites of hydroxyapatite (HAp) and Gum arabic were prepared and evaluated by various spectroscopic and analytical techniques. Hydroxyapatite and GA composite is bio-based and have unique properties such as biocompatibility, bioactivity and osteo-conductivity. These properties make it attractive various applications such as metals extractions. The composite was prepared by the solution method. The possibility of using the prepared composite as a based stationary phase for removal of arsenic (III) from waste water was evaluated. The composite offered in this work could be a very promising adsorbent for arsenic (III).

## Methods

### Materials

Gum Arabic (GA) was obtained from the southern area of Morocco: Laayoune-Smara. The Ca(NO_3_)_2_*4H_2_O (99%), (NH_4_)_2_HPO_4_ (99%) were purchased from Aldrich in high purity forms and used as re. Muller-Hinton as received. (Biokar); Muller-Hinton broth (Biokar); potato dextrose agar (PDA), sterile distilled water, and sterile paper discs were used in this work. All synthesis and testing procedure were carried out in triplicates.

### Synthesis of HAp/GA composite

**Fig. 1:**
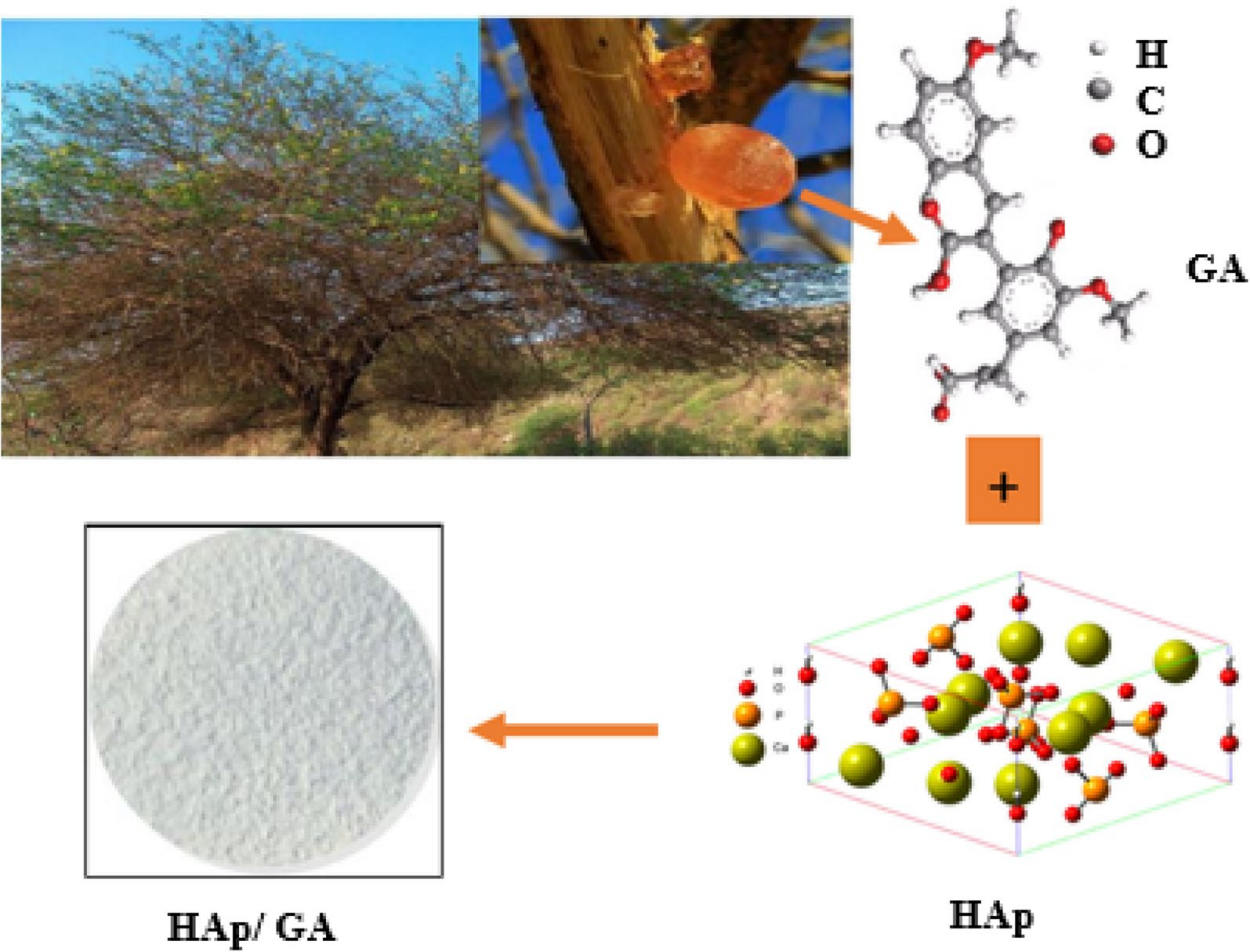
Schematic representation of synthesis route HAp/GA composite

The HAp/GA composites were prepared using various ratios of HAp and GA as shown in Table 1. The general procedure for making the composites is as follows: an aqueous solution of Ca(NO_3_)_2_·4H_2_O (11.76 g) was added drop-wise to an aqueous solution of (NH_4_)_2_·HPO_4_ (4.06 g), with stirring. The molar ratio of calcium to phosphorous was about 1.67. Then GA was added to the solution in an amount equal to 10% by weight of the two materials, followed by a dropwise addition of ammonium solution (25%) to adjust the pH of the reaction solution to 10.5. The reaction mixture was heated to 90 °C and maintained at this temperature for 1 h. The reaction was then cooled down and stirred at room temperature for 120 min. The resulting precipitate was filtered and dried in an oven at 50 °C to produce a fine powder [4] as shown in Fig. 1.

**Table 1:** Quantities of reagents used in the preparation of the composite

	HAp (W)	GA (W)
A	50	50
B	60	40
C	70	30

### Chemical structure

#### Characterization of the composite

The produced composite was analyzed by infrared spectroscopy (ATR FT-IR), using a Schimadzu FT-IR 300 series instrument (Shimadzu Scientific Instruments). FTIR spectra were acquired over the region 400–4000 cm^−1^. 1.0 mg of powder samples were mixed with 200.0 mg of KBr (spectroscopic grade) using a mortar, then pressed to form a pellet. The composite structure was also evaluated by X-ray diffraction (XRD) using a Rich Siefert 3000 diffractometer (Seifert, Germany) with Cu–K [(Seifert, Germany) wi8A]. Emission scanning electron microscopy (SEM) was used to investigate the morphology of the prepared composites and the filler/matrix interface by using an SU 8020, 3.0 kV SE(U).

#### Swelling and biodegradability of the composites

Swelling and biodegradability of the composites were studied by immersing a known weight of the composite HAp/GA (W_0_) in a solution of biological medium PBS (10 mL) at 37 °C. The fluid was buffered to the physiological pH of 7.4. The swelling behavior was evaluated over 1–24 h period. The wet sample was weighed (W_1_) then dried at 40 °C for 30 min and weighed to produce the final weight (W_f_). The water absorption capacity (expressed in percentage) was calculated by subtracting the initial weight (W_0_) from wet weight (W_1_) and dividing over the initial weight as shown in Eq. (1).

1 $$Water\;absorption = \frac{{\left( {W_{1} - W_{0} } \right)}}{{W_{0} }}*100$$

The mass loss was calculated according to Eq. (2)

2 $$Weight\;loss\;\left( \% \right) = \frac{{\left( {W_{f} - W_{0} } \right)}}{{W_{0} }}*100$$

#### Adsorption of arsenic

The experiment was carried out in a polyethylene beaker that was rinsed with ultrapure water. To the beaker was added an aqueous solution of arsenic with various concentrations (2, 5 and 10 mg/L). To the solution in the beaker was added a sample of the composite (200.0 mg). The produced mixture was stirred for various time periods (15, 30, 45, 60, 120, 180 and 240 min). The mixture was the filtered through a glass funnel fitted with a filter paper and rinsed with ultrapure water. The filtrate from the rinse (50 mL) was collected in a separate test tube and acidified with 500 μL of pure nitric acid. The produced acidic solution was subjected to analysis by Atomic Emission Spectrometry (ICP, AES Ultima 2-JobinYvon). The beak area represents the arsenic was used to determine the concentration of arsenic from a pre-prepared calibration curve.

### Adsorption experiments and kinetic parameter

#### Process of adsorption

The composite adsorption capacity (Qe) of resin was calculated by Eq. (3) [28]:

3 $$Q_{e} = \frac{{\left( {C_{0} - C_{e} } \right)V}}{W}$$

where Q_e_ is the amount of metal ions adsorbed (mg arsenic/g composite), C_0_ is the initial concentration of As (III) ion in ppm, Ce is the final concentration of As (III) ion in ppm; V is the volume of As (III) ion solution (mL) and W is the weight of the composite (g).

#### Adsorption isotherms

##### Langmuir isotherm

Langmuir isotherm was calculated according to Eq. (4) [28]:

4 $$\frac{{C_{e} }}{{Q_{e} }} = \frac{{C_{e} }}{{Q_{m} }} + \frac{1}{{Q_{m} b}}$$

where C_e_ is the final concentration of arsenic (ppm), Q_e_ is the amount ometal ions adsorbed by the composite (mg/g), Q_m_ is the maximum amount of adsorption of metal ions (mg/g), and b is the adsorption equilibrium constant of Langmuir (mL/mg). Equation (4) is a straight line equation, so plotting C_e_/Q_e_ versus C_e_ produces a straight line with a slope equal to 1/Q_m_ and an intercept of 1/(Q_m_b).

##### Freundlich isotherm

Freundlich isotherm is shown Eq. (5) [28]:

5 $$\ln Qe = b_{F} \ln C_{e} + \ln K_{F}$$

where C_e_ is the final concentration of arsenic (ppm), Q_e_ is the amount of metal ions adsorbed by the composite (mg/g), K_F_ is the maximum amount of adsorption of metal ions (mg/g) and b_F_ is the adsorption intensity. K_F_ and b_F_ are constants, Freundlich was determined by plotting lnQ_e_ versus lnC_e_.

##### Isotherm Dubinin–Radushkevich

The isotherm Dubinin–Radushkevich shown in Eq. (6) has an important use, since it distinguishes between physical and chemical adsorption [28]:

6 $$\ln Qe = K\varepsilon^{2} + \ln Q_{DR}$$

where Q_e_ is the amount of metal ions adsorbed (mg/g), Q_DR_ is the maximum adsorption capacity of metal ions (mg/g), K is the Dubinin–Radushkevich constant (kJ^2^/mol) and ε is Polanyi potential usually calculated according to Eq. (7) [28]:

7 $$\varepsilon = RT \ln \left(1 + \frac{1}{{C_{e} }}\right)$$

where C_e_ is the final concentration of arsenic (ppm), R is the ideal gas constant (J/mole K) and T the temperature in K. Plotting ln Q_e_ against ε^2^ gives a straight line with a slope equal to K and intercept QDR. Inserting the value of the constant Dubinin–Radushkevich obtained from Eq. (7) in Eq. (8) gives average adsorption energy [28]:

8 $$E = 2K^{ - 1/2}$$

where E is the average adsorption energy (kJ/mol), and K is the constant Dubinin–Radushkevich.

#### Kinetic parameter

The monomolecular reaction is a first order reaction that depends on the concentration of a single compound, usually written as shown in Eq. (9) [10]:

9 $$As(aq) + HAP - GA(s) \to HAP - GA - As(s)$$

where As(aq) represents the arsenic in the aqueous phase, HAP/GA (s) is the available reactive surface of the media for arsenic adsorption. HAP/GA-As (s) is the concentration of Arsenic in the composite and k_ads_ is the adsorption reaction rate constant, which can be represented as shown in Eq. (10):

10 $${\text{K}}_{\text{ads}} = \frac{[HAP - GA - As\left( s \right)]}{{[As\left( {aq} \right)][HAP - GA\left( s \right)]}}$$

According to Eqs. (9) and (10), the reaction rate equation becomes (Eq. 11):

11 $$\frac{{d\left[ {As\left( s \right)} \right]}}{dt} = - {\text{Kads}}\frac{{[HAP - GA - As\left( s \right)]^{a} }}{{[HAP - GA (s)]^{b} }}$$

where [] is the molar concentration of As, ‘‘a’’ and ‘‘b’’ are the order(s) of reaction, and “t” is the adsorption time.

##### Kinetic models of arsenic (III) adsorption

The pseudo first-order model:

The pseudo-first order equation representing the curve of log(Q_t_ − Q_e_) versus time could be written as shown in Eq. (12):

12 $$Log\left( {Q_{e} - Q_{t} } \right) = Log\left( {Q_{e} } \right) - \frac{kt}{2.303}$$

where Q_t_ is the amount of arsenic adsorbed at time t in mg/g, Q_e_ is the amount of arsenic adsorbed at equilibrium (mg/g), and k is the initial adsorption rate (min^−1^).

The pseudo second-order model:

The pseudo second-order model could be used to predict the kinetic parameters of the linear equation, it can be written as Eq. (13):

13 $$\frac{t}{Q}_{t} = \frac{1}{{k^{\prime}Q_{e}^{2} }} + \frac{1}{{Q_{e} t}}$$

14 $$h = k^{\prime}Q_{e}^{2}$$

where Q_t_ is the amount of arsenic adsorbed at time t (mg/g), Q_e_ is the adsorption capacity of arsenic adsorbed at equilibrium (mg/g), k′ is the equilibrium rate constant of pseudo-second order (g/mg min), h is the initial sorption rate (mg/g min).

Intra-particle diffusion model:

This model is controlled by the diffusion step. The amount adsorbed Q_e_ is directly proportional to the square root of time t as shown in Eq. (15). [10]:

15 $$Q_{e} = k_{i} t^{{\frac{1}{2}}}$$

where Q_e_ is the amount of arsenic adsorbed at time t, k_i_ is the intra-particle rate constant (mg/g min^1/2^).

### Antibacterial and antifungal tests

This study was carried out using the disc diffusion method using three bacterial strains *Micrococcus luteus*, *E. coli* and *Bacillus subtilis*.

The Disc diffusion method for antimicrobial susceptibility testing was carried out according to a standard method by Bauer et al. [29] to assess the presence of antibacterial activities of the Hap/GA composite. A bacteria culture (which has been adjusted to 0.5 McFarland standard), was used to lawn Muller Hinton agar plates evenly using a sterile swab. The plates were dried for 15 min and then used for the sensitivity test. To the discs were added known weight of HAp/GA composite powder and placed on the Mueller–Hinton agar surface. Each test plate comprises of six discs: A positive control (Tetracycline 1 mg/mL), a negative control (DMSO), and four treated discs. All plate discs were placed in a plate about equidistant to each other. The plate was then incubated for a period of time depends on bacteria cell type *M. luteus* and *E. coli* were incubated at 37 °C and at *B. subtilis* at 33 °C for 18 to 24 h. On the other side, the plate of the fungi Candida albicans contained PDA (potato dextrose agar) was incubated at 37 °C for 48 h, cycloheximide was utilized as an antifungal control. After incubation, the inhibition zone was measured using a caliper. The test was repeated three times to ensure reliability.

## Results and discussion

### FTIR analysis

**Fig. 2:**
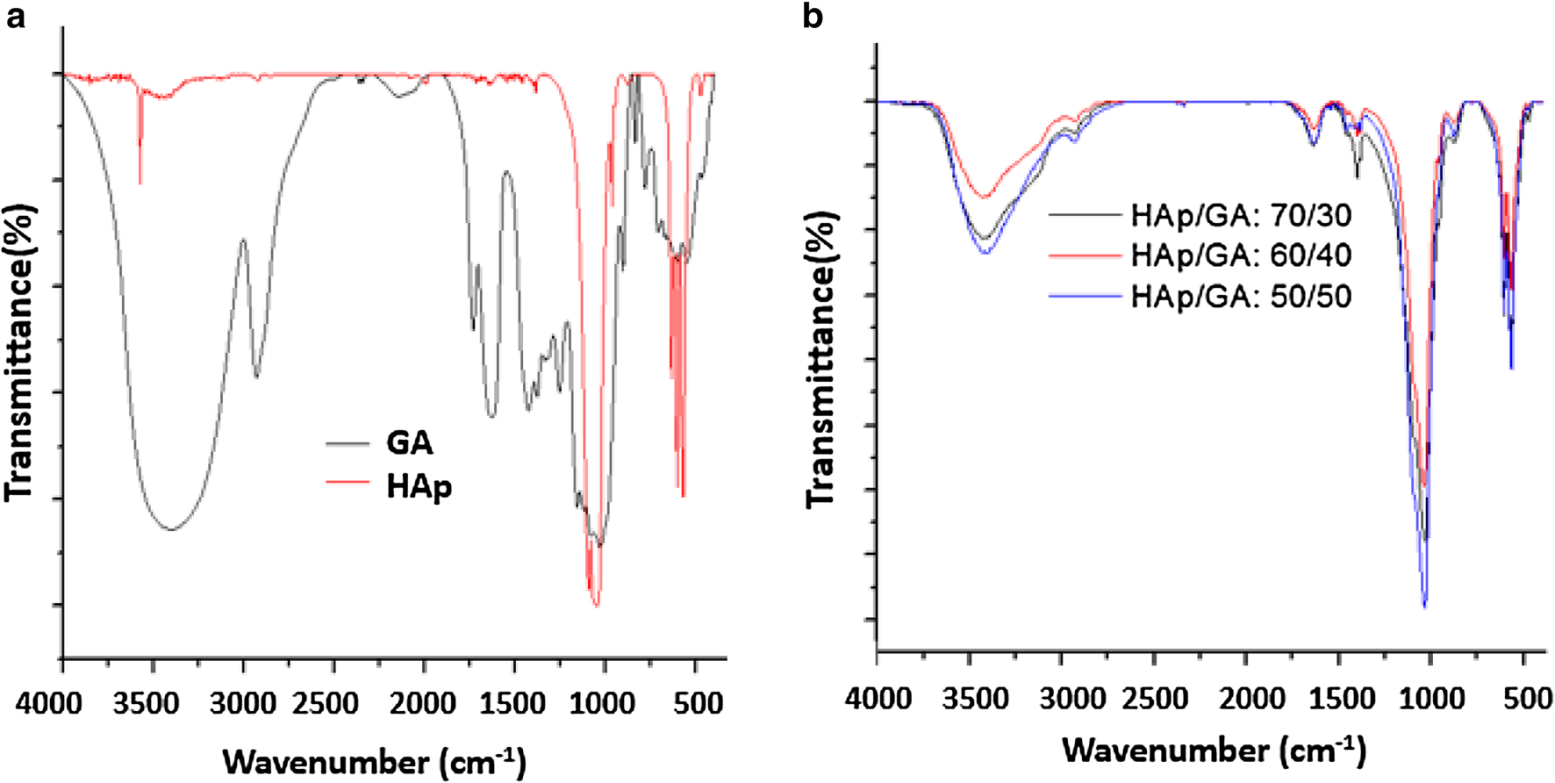
**a** FTIR spectra of HAp and Gum Arabic. **b** The infrared spectra of the HAp/GA composite

The structures of the HAp, GA and HAp/GA composite were analyzed by FT-IR spectroscopy, obtained spectra are shown in Fig. 2. The IR spectra of GA and HAp are overlaid in Fig. 2a. the IR shows a band at 3419 cm^−1^ corresponds to the OH stretching vibration of the Arabic gum. A band also appears at 2932 cm^−1^ corresponding to the C–H stretching. The peaks at 1600 and 1420 cm^−1^ could be attributed to the asymmetric and symmetric stretching vibrations of the carboxylate COO^–^ group. The stretching vibrations of ether C–O–C and hydroxyl C–O of carboxylate appear at 1135 and 1073 cm^−1,^ respectively. A smaller band of the glycosidic bonds appear as a week band at 896 cm^−1^.

The IR spectrum of HAp is shown in Fig. 2a. The spectrum shows the presence of a band at 3400 cm^−1^ which corresponds to the OH bond vibration. The bands shows between 1100–900 cm^−1^ (especially the bands located at 1090, 1050 and 962 cm^−1^) and 600–500 cm^−1^ (particularly the bands located at 603 and 571 cm^−1^) could be attributed to PO_4_^3−^ apatitic [30].

The FT-IR of the HAp/GA composite (Fig. 2b) shows a band near 1683 cm^−1^ which could be related to the CO stretching vibration. The peaks at 1420 cm^−1^ could be assigned to the asymmetric and symmetric stretching vibrations of the carboxylate group. The interaction between the COOH of GA and OH of HAp is probably responsible for the appearance of this new very low bandwidth. In addition, the composite IR spectrum shows an absorption band at 3550 cm^−1^ corresponding to the hydroxyl group.

### XRD analysis

**Fig. 3:**
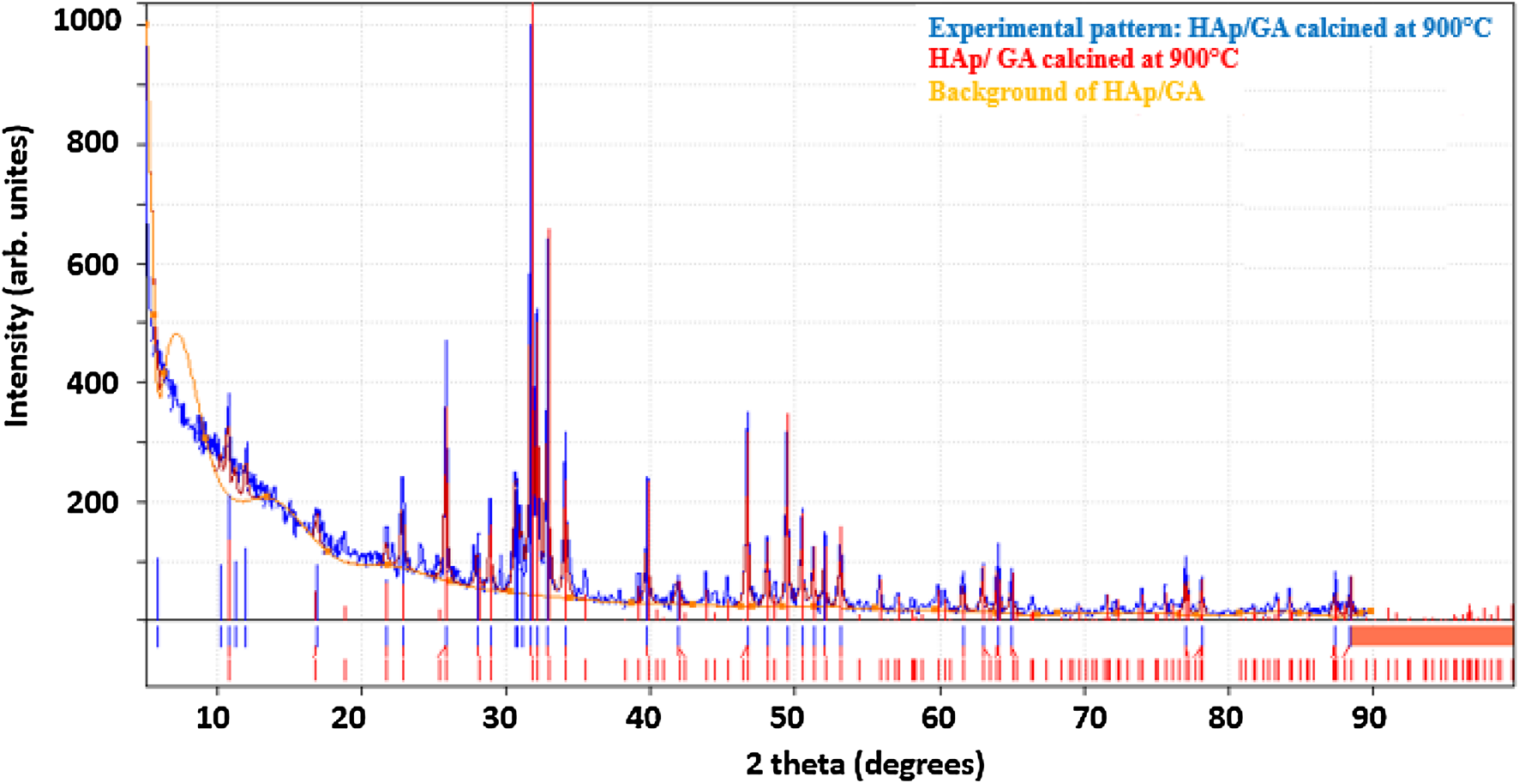
XDR patterns of HAp/GA composite calined at 900 °C

The based composite HAp and GA was calcined at 900 °C. At this high temperature the organic matrix burned completely, so their hydroxyapatite is only left to be analyzed by XRD. The X-ray patterns collected on the powders after heat treatment at 900 °C for 2 h presented a single phase of HAp. No characteristic peaks of impurities such as calcium hydroxide and calcium phosphate were observed. This indicate that, pure HAp was prepared under the present experimental condition. The diffraction peaks particularly in the planes (002), (211) and (300) were high and narrow indicating that HAp has a crystalline structure (Fig. 3).

**Fig. 4:**
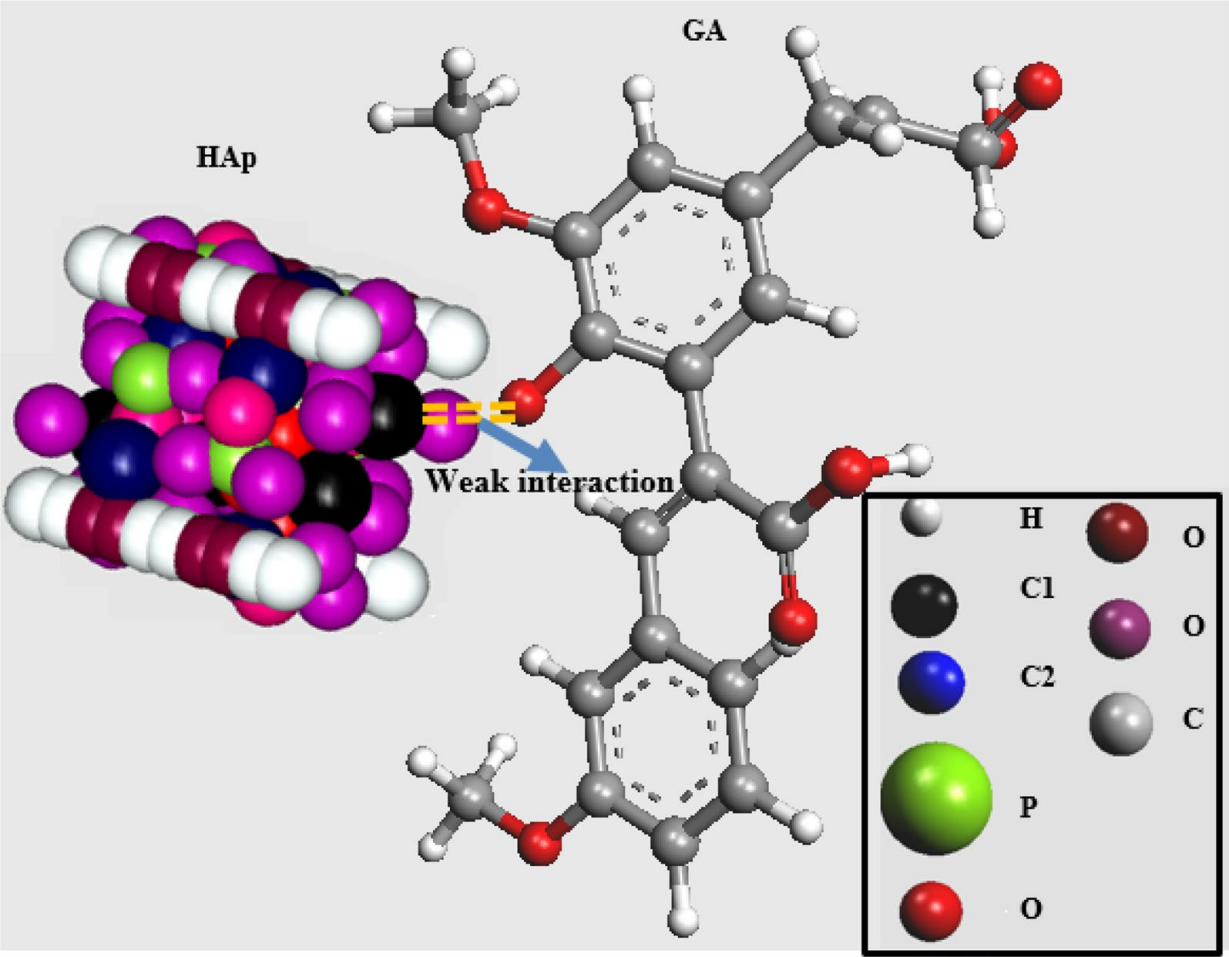
A 3D schematic model of the weak interaction between the CO groups in GA and the HAp

Based on the FT-IR results, a model that represents the hydrogen bonding between CO groups in GA and the OH groups in HAp μ-particles is depicted in Fig. 4. The GA polymer chains are randomly twisted and inhibit the reversible phase during the transition from glassy state to rubbery state. The model may also be used to explain the outcome of FT-IR results.

### Microscopic observation SEM

**Fig. 5:**
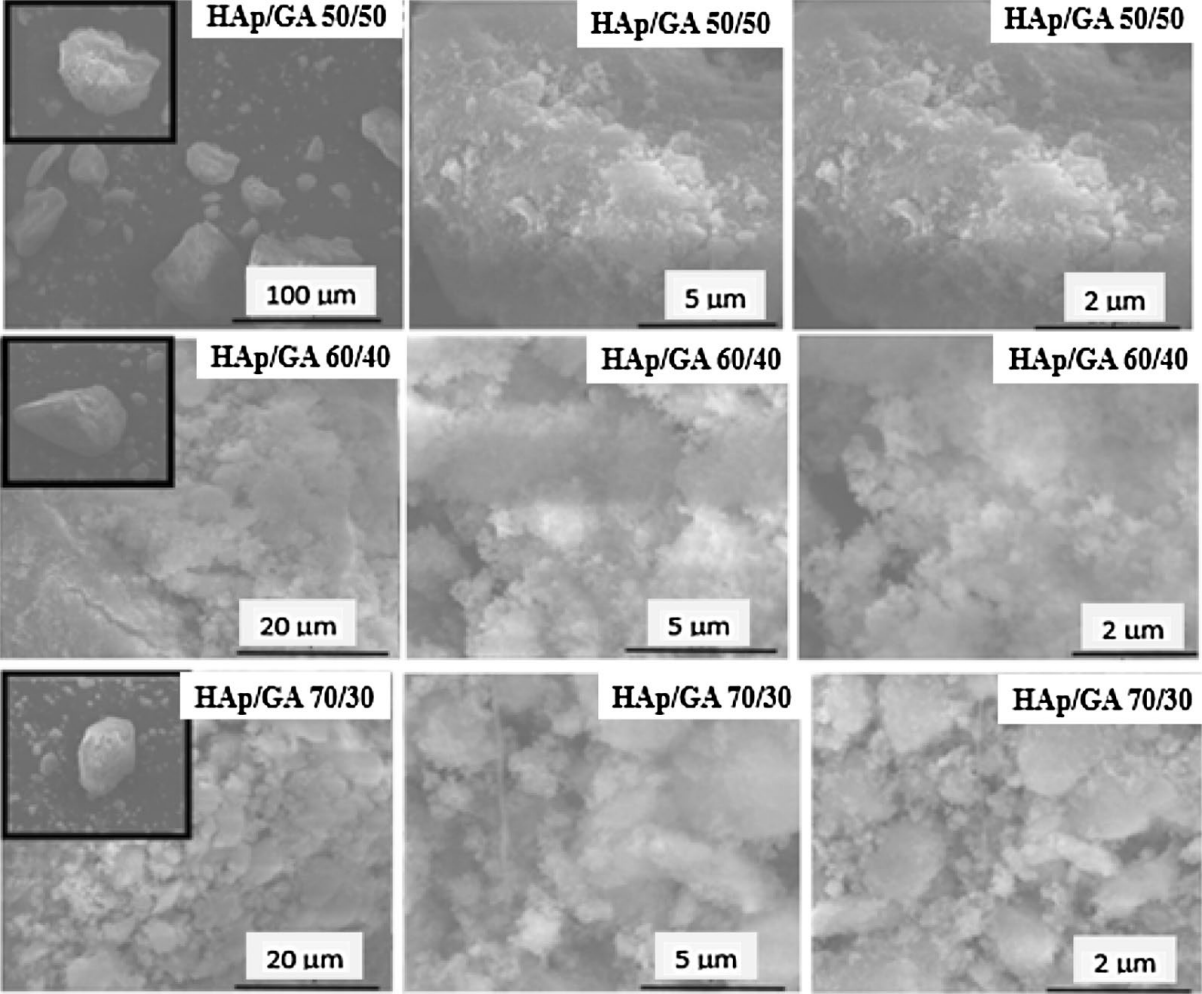
The SEM of HAp and HAp/GA composite scaffold

The SEM images of HAp/GA composite are shown in Fig. 5. The images show clearly the morphology and distribution of the grains in the composite. The HAp/GA composite image shows that HAp crystals are still in the range of a μ-meter scale and have a good dispersive property all over the composite structure. The image of the HAp/GA composite also discloses that, the scaffold was a three-dimensional irregular porous structure, assembled together with clear interconnections between the pores. The macro pores contained many microspores.

### Swelling and biodegradability of HAp/GA composite

**Fig. 6:**
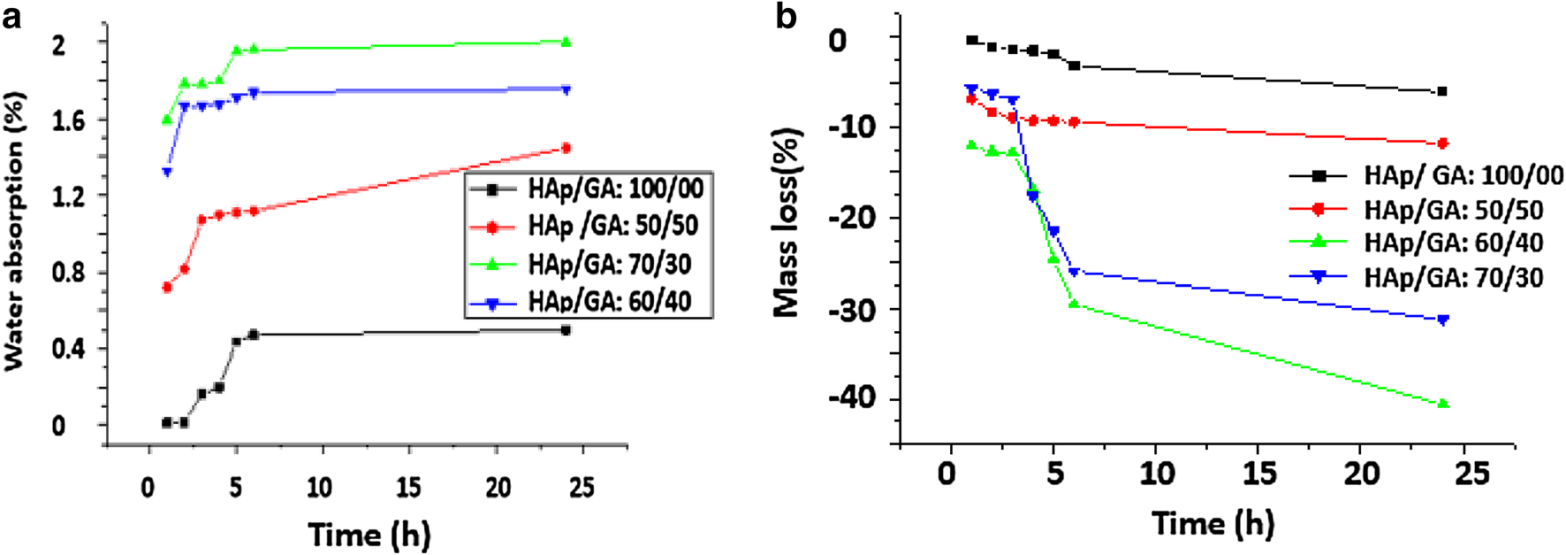
The rate of weight loss (**a**) and water absorption (**b**) as a function of soaking time

The rates of weight loss and water absorption of three HAp/GA composites as a function of time were evaluated in PBS solution with a pH of 7.4 at 37 °C. The results are plotted in Fig. 6.

The results show that, the loss in the weight of the composite increased by increasing the amount of HAp in the composite. The surface became coarser, more porous and absorbed more water. Figure 6a clearly shows that, the water absorption and the rate of degradation of the composite materials increased by increasing HAp content. The weight loss of the composite HAp/GA immersed in PBS were as follows: after 1 h of immersion the weight loss of the composite HAp/GA with 70/30 was about 12.61%. Composite with a 50/50 composition showed a weight loss of 11.81% after 24 h of immersion. The 70/30 composites showed a loss of 41.56%, and the 60/40 composite showed a loss of 31.92%. Composites with 70% HAp and 30% GA lost about 41.56% of their weights after 24 h, then a slight increasing in mass was noticed (Fig. 6b).

### Total organic carbon production

**Fig. 7:**
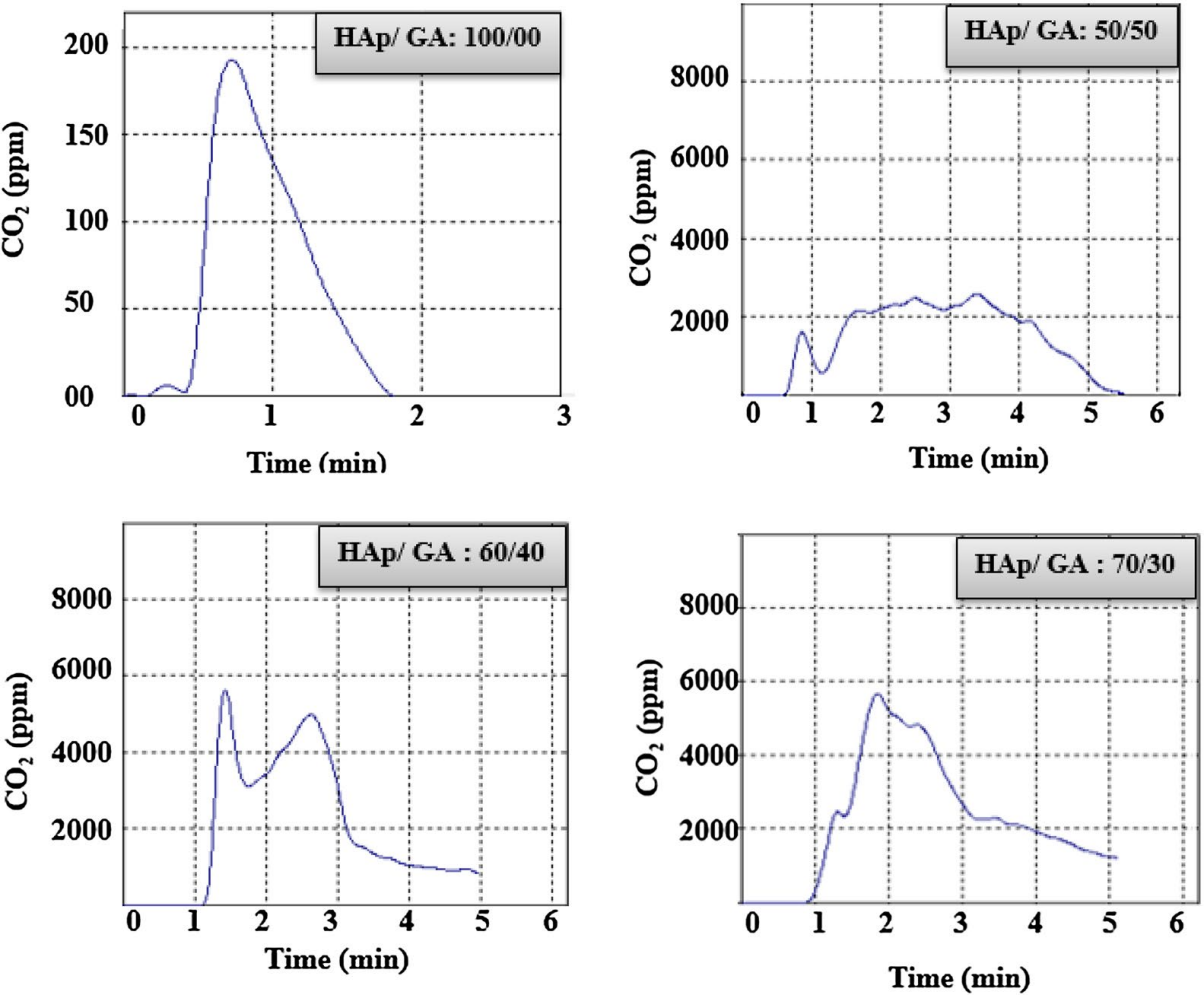
Plot of TOC versus time for HAp and HAp/GA composite scaffold

Results of TOC are shown in Fig. 7. The results indicated that carbon production for GA is higher than that produced by the composites. As shown in Fig. 7, the TOC results show that, composites with 50% HAp produced lower CO_2_. The TOC level of composites was controlled by the % of HAp in the composite, the higher the HAp content the lower the CO_2_ production. This could be an indication that, the interaction between GA and HAp increases by increasing the HAp content.

### Adsorption isotherms

**Fig. 8:**
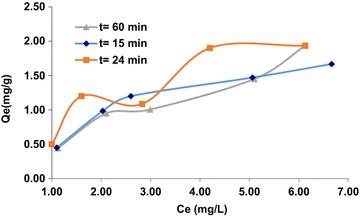
Adsorption isotherms of arsenic (III) ion on the composite HAp/GA at t = 15 min, t = 24 min and t = 60 min

The results of analysis of inductively coupled plasma (ICP) are plotted in Fig. 8.

**Fig. 9:**
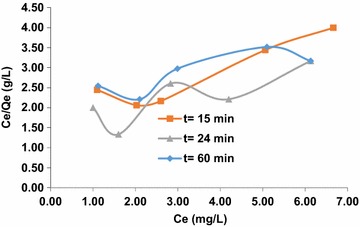
Adsorption isotherms of arsenic (III) ion on the composite HAp/GA at t = 15 min, t = 24 min and t = 60 min, linearized according to Langmuir

**Fig. 10:**
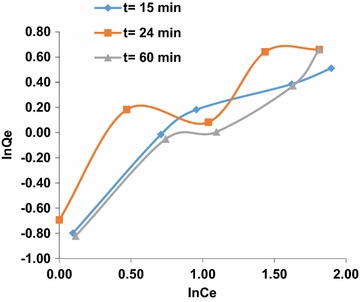
Adsorption isotherms of arsenic (III) ion on the composite HAp/GA at t = 15 min, t = 24 min and t = 60 min, linearized according to Freundlich

**Fig. 11:**
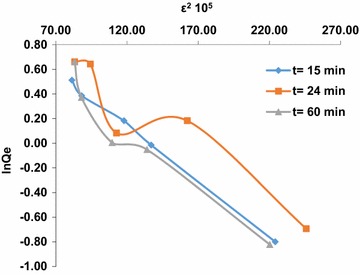
Adsorption isotherms of arsenic (III) ion on the composite HAp/GA at t = 15 min, t = 24 min and t = 60 min, linearized according to Dubinin–Radushkevich equations

Graphic representations of the isotherm equations were used to study the adsorption parameters. The plotting results show that, the correlation coefficient at t = 15 min in the equation of the Dubinin–Radushkevich isotherm is greater than the value of the coefficient (R^2^) of the Langmuir equation and Freundlich equation. This indicates that, the Dubinin–Radushkevich model is more suitable for the description of the adsorption of arsenic (III) into the HAp/GA composite. The results show that, the interaction between adsorbent and adsorbate is physiochemical (physical and chemical). The values of K and QDR are obtained from Dubinin–Radushkevich isotherm to be −0.009 kJ^2^/mol and 3.0925 mg/g, respectively with R^2^ equal to 0.9974. The results indicate that in the first 15 min of contact between the arsenic solution and the composite most of the active sites on the composite surface area were vacant, and a little adsorption occurs. As time goes on, the adsorption sites become more saturated as shown in Fig. 9. After 15 min of contact time, it was noticed that the linearity of adsorption isotherm models depended on the concentration of arsenic (III). The correlation coefficient (R^2^) of linear Freundlich model is superior to the Dubinin–Radushkevich isotherm and the Langmuir equation. Also, the amount adsorbed increased with increasing the concentration of arsenic (III). While the composite bonding sites became more saturated, the physical adsorption isotherm Dubinin–Radushkevich was dominated at the first 15 min. The values of KF and BF were obtained from Freundlich isotherms to be 0.4388 and 0.794, respectively with R^2^ equal to 0.9562. The model assumes an infinite Freundlich occupation adsorbents sites that vacantly tend to represent heterogeneous elements [31] as shown in Figs. 10 and 11.

After 24 h of contact time between arsenic (III) solution and the composite HAp/GA, it was found that, the coefficient of the equation of the isotherm Dubinin–Radushkevich is greater than the coefficient values (R^2^) obtained from Freundlich equation ETDE Langmuir. The values of K and QDR were obtained from Dubinin–Radushkevich isotherm and were equal respectively to −0.0079 kJ^2^/mole and 3.5243 mg/g, with R^2^ equal to 0.8831. Therefore, the Dubinin–Radushkevich model is reversible, which implies that the saturation composite sites of HAp/GA by the As(III) ions is complete. These results indicate as shown above that, the interactions between the composite HAp/GA and the adsorbate is a physical–chemical.

### Kinetics effect

**Fig. 12:**
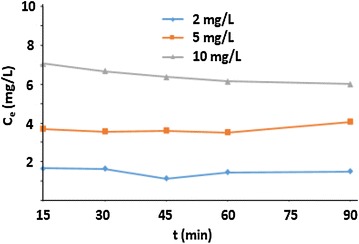
kinetic adsorption of Arsenic (III) at 2, 5 and 10 mg/L

**Fig. 13:**
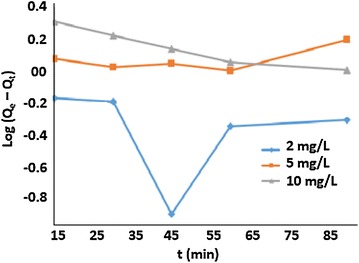
Plot of pseudo first order kinetic modelat 2, 5 and 10 mg/L

The kinetics and the concentration of arsenic (III) on the composite HAp/GA rate of adsorption were studied. The curve of Ce (mg/L) versus time show that, the concentration of arsenic (III) stayed constant during the experiment [32]. It was also found that, the concentration of the adsorbent does not have an effect on the reaction kinetics. This could be attributed to both the size difference between the composite molecule and the metal ion, and to the physical–chemical interactions as shown in (Figs. 12, 13).

#### Kinetic models of arsenic (III)

**Fig. 14:**
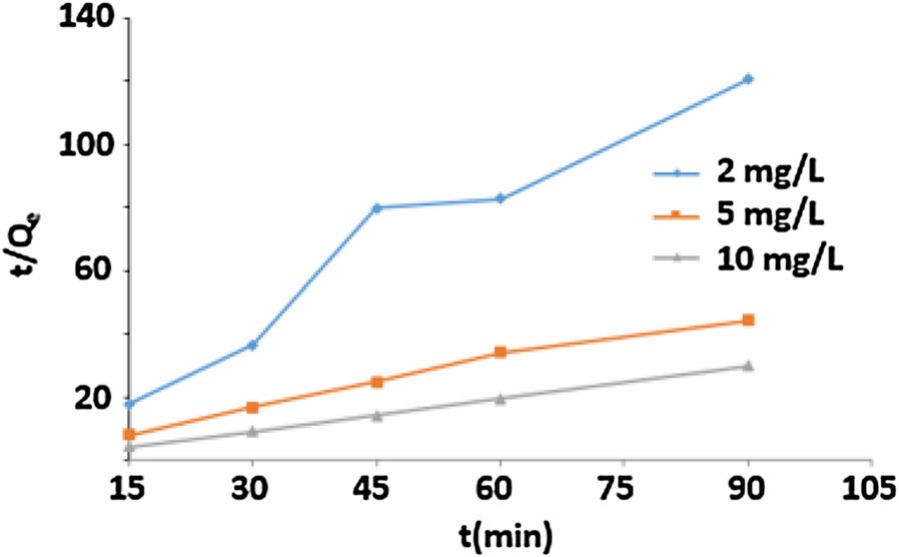
Plot of second first order kinetic model at 2, 5 and 10 mg/L

The variation of $t/Qe$ as a function of time for the arsenic (III) solutions with concentrations of 2, 5 and 10 mg/L is depicted in Fig. 14. It was observed that, when the metal concentration increases, the line becomes linear. The effect of the amount of arsenic (III) ions played an important role on the process of adsorption, which could be due to the large number of available active sites. The correlation coefficient of the 10 mg/L solution is R^2^ > 0.93. The assumed rate of adsorption is proportional to the difference between the amount of arsenic adsorbed at equilibrium (Q) and the amount of arsenic adsorbed as a function of time, which is represented by Q_t_ [11]. The adsorption mechanism was studied by the second-order model, results are shown in Fig. 14, the correlation coefficients were determined to be greater than 0.98 for the concentration of 5 and 10 mg/L. These results explain the first model results, and show that, a greater amount of adsorbate increased the reliability of the experiment. Correlation coefficient T/Q_t_ as a function of time proves that, the reaction is a second order. Therefore, the sorption system is limited by a chemical adsorption [32].

#### The characteristics of adsorption surface

**Fig. 15:**
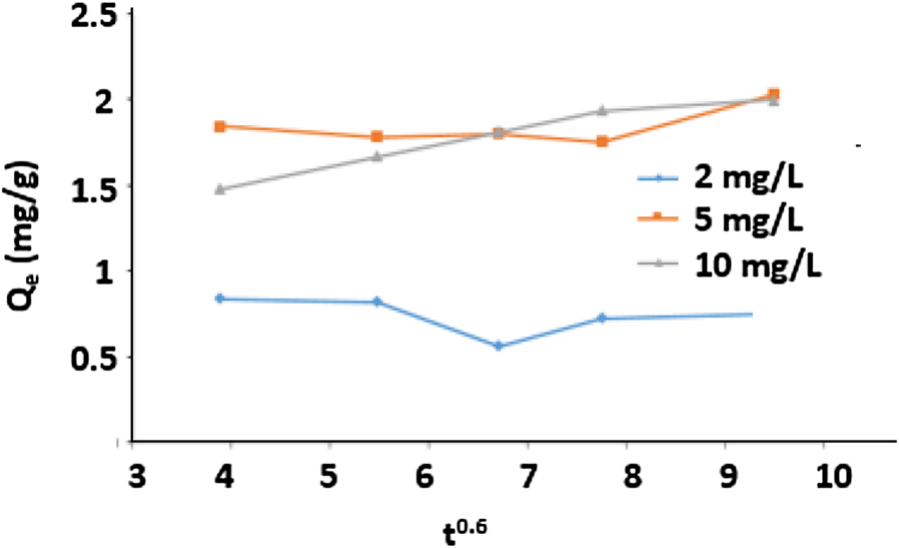
Intraparticle diffusion model (Weber–Morris) for As (III) at 2, 5 and 10 mg/L

The diffusion model (Weber–Morris) was used to study the characteristics of the adsorption surface. Figure 15 shows the intra particle model (Weber-Morris) for 2, 5 and 10 mg/L solutions of arsenic (III). The migration of the adsorbate from the solution to the composite sites was characterized by diffusion or by intra-particle diffusion (mass transfer through the pores), or could be a combination of both [33]. The diffusion pattern indicated the formation of chemical bonds between adsorbate and adsorbent.

### Antibacterial and antifungal test

**Fig. 16:**
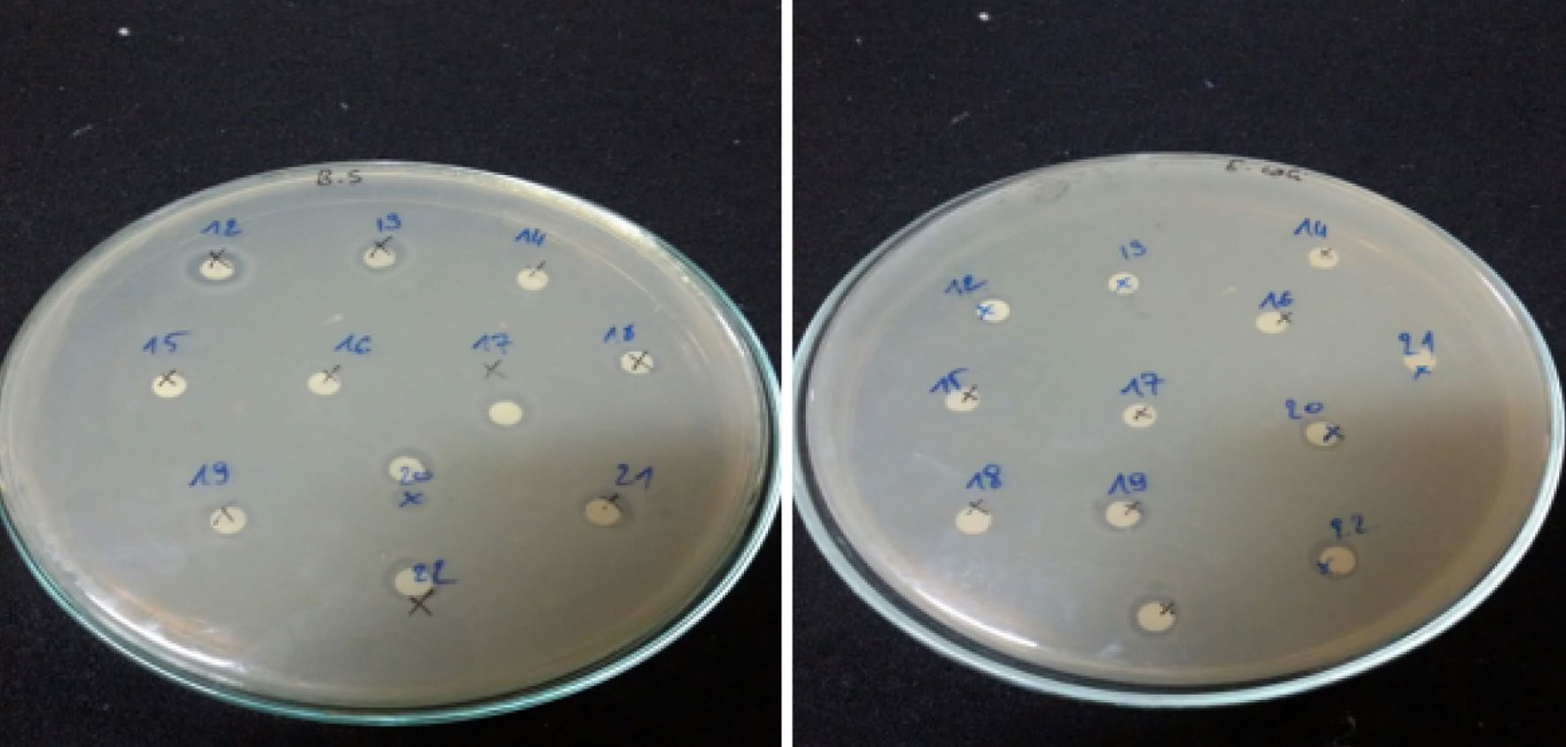
Sensitivity test in agar media

The HAp/GA composites were evaluated for antimicrobial activities. The sensitivity test results showed that, the rate of inhibition is affected by the composite components ratios. Results are shown in (Fig. 16) and in Table 2. Composite III (HAp/GA 60/40) was found to inhibit the growth of *B. subtilis* (B.S) with a diameter of inhibition of about 8.0 mm and showed a major inhibition of *M. luteus* (11.0 mm, M. L).

**Table 2:** Diameter of inhibition (mm) of the prepared composites tested on three bacteria and one fungus

Stains	50/50	60/40	70/30	C+
*B.S*	3	8	4	25
*M.L*	5	11	6	25
*E. coli*	–	–	–	26
*Candida*	3	6	2	25

The composite (HAp/GA 60/40) showed also antifungal activity with a diameter of inhibition on Candida albicans of about 6.0 mm. The composites showed a lower diameter of inhibition when compared to the positive control, which showed 25.0 mm for *M. luteus*, 25.0 mm for *B. subtilis*, 26.0 mm for *E. coli*, and 25.0 mm for Candida.

All other composites (50/50, 60/40, 70/30 and HAp) didn’t show any activity against the tested bacterial strains and fungi.

## Conclusion

Several HAp/GA composites with various weight ratios were prepared by the solution method. The prepared composites were evaluated by various spectroscopic and analytical methods such as Fourier transform infrared spectroscopy (FT-IR) and scanning electron microscope (SEM). The analysis results showed that, the interaction between the components of the composite was facilitated by H-bonding. The prepared composites were designed to extract the toxic metal arsenic (III) from an aqueous solution. The effects of arsenic concentration, contact time (t) and the complexing nature of HAp/GA composite on the adsorption rate arsenic (III) were evaluated. The three adsorption isotherms: Langmuir, Freundlich and Dubinin Radushkevich were applied to study the mechanism involved in the adsorption of arsenic (III) by the composite. The adsorption kinetic showed that, the adsorption of arsenic (III) on the HAp/GA composite was controlled by two main factors the initial concentration of arsenic (III) and the contact time. The kinetic studies showed that, the rate of adsorption of arsenic (III) by the composites is a second order. Results further showed that some of the HAp/GA composites have activities against antimicrobial and antifungal. The composites offered in this study could be a valuable approach for removing toxic metals for contaminated water.
